# Effective Temporal Graph Learning via Personalized PageRank

**DOI:** 10.3390/e26070588

**Published:** 2024-07-10

**Authors:** Ziyu Liao, Tao Liu, Yue He, Longlong Lin

**Affiliations:** College of Computer and Information Science, Southwest University, Chongqing 400715, China; lzy2021xndx@email.swu.edu.cn (Z.L.); taoliu.swu@gmail.com (T.L.); estherr@email.swu.edu.cn (Y.H.)

**Keywords:** dynamic graph representation, time-constrained personalized PageRank, matrix factorization, link prediction, node classification

## Abstract

Graph representation learning aims to map nodes or edges within a graph using low-dimensional vectors, while preserving as much topological information as possible. During past decades, numerous algorithms for graph representation learning have emerged. Among them, proximity matrix representation methods have been shown to exhibit excellent performance in experiments and scale to large graphs with millions of nodes. However, with the rapid development of the Internet, information interactions are happening at the scale of billions every moment. Most methods for similarity matrix factorization still focus on static graphs, leading to incomplete similarity descriptions and low embedding quality. To enhance the embedding quality of temporal graph learning, we propose a temporal graph representation learning model based on the matrix factorization of Time-constrained Personalize PageRank (TPPR) matrices. TPPR, an extension of personalized PageRank (PPR) that incorporates temporal information, better captures node similarities in temporal graphs. Based on this, we use Single Value Decomposition or Nonnegative Matrix Factorization to decompose TPPR matrices to obtain embedding vectors for each node. Through experiments on tasks such as link prediction, node classification, and node clustering across multiple temporal graphs, as well as a comparison with various experimental methods, we find that graph representation learning algorithms based on TPPR matrix factorization achieve overall outstanding scores on multiple temporal datasets, highlighting their effectiveness.

## 1. Introduction

Graph embedding refers to the method of representing the structural attributes of a graph using low-dimensional vectors [1]. This approach transforms complex network structural information into compact vector representations, facilitating analysis, processing, and application of graph structural information across various tasks like node classification [2], link prediction [3], and graph reconstruction [4]. Various technical methods have been developed for graph embedding, broadly categorized into traditional methods and those based on graph neural networks. These methods aim to derive low-dimensional vector representations from graphs. Additionally, similarity matrix factorization techniques have shown promising experimental results [5]. While previous research has predominantly focused on static graphs, today’s online environments are characterized by stacked temporal graphs. Consequently, there is a pressing need for algorithms capable of accurately and efficiently characterizing temporal structures. Current approaches in temporal attribute graph representation learning face challenges. For example, Temporal Graph Convolutional Networks (T-GCN) [6] are limited to handling temporal attribute graph data with fixed topological structures and struggle to adapt to changing topologies. Dynamic Graph Convolutional Networks (D-GCN) [7], on the other hand, are suitable only for labeled data, which is not always practical due to high labeling costs. Traditional graph deep learning models like Graph Convolutional Networks (GCN) [8] often suffer from over-smoothing issues, hindering effective feature extraction from networks.

**Figure 1:**
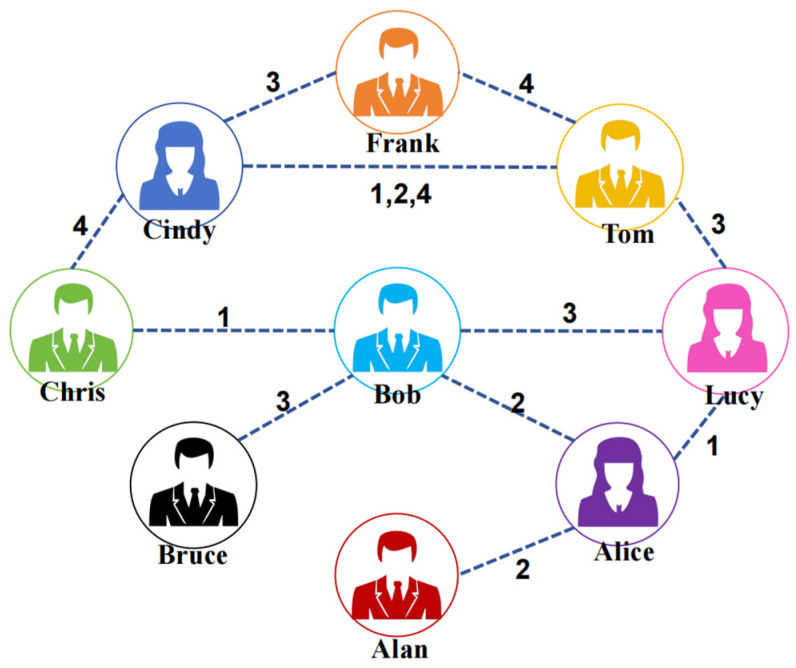
Dynamic local structural graph.

In graph embedding learning, Personalized PageRank (PPR) [9] is also a key focus. PPR aims to learn low-dimensional representations of nodes in a graph while preserving their similarities, better characterizing the structural features of the graph. However, existing PPR-based graph embedding learning methods are mostly applied to static graphs, such as HOPE [10], VERSE [11], and some recent works, which also extend on PPR. Therefore, research on PPR in dynamic graphs is still incomplete, facing the following major challenges: (i) Without constraints of time attribute, the random walk sequence <Alice, Lucy, Tom, Frank> is reasonable in a community contact graph, shown in Figure 1. However, with added time attributes as shown in Figure 1, interactions occur at different timestamps, which makes the random walk sequence <Alice, Lucy, Tom, Frank> unreasonable as a temporal random walk sequence. (ii) In Figure 1, based on the timestamps of interactions, it is obvious that the likelihood of the temporal walk edge <Chris, Bob, 1> transitioning to <Bob, Alice, 2> in the next timestamp is greater than transitioning to <Bob, Lucy, 3>. However, such transition probabilities cannot be reflected in traditional PPR transition matrices. (iii) Due to the dynamics of temporal graphs, edges that are connected at one timestamp may not be reachable at the next timestamp, which is known as the dangling edge problem.

To address the challenges mentioned above, we found that the key lies in how to represent temporal node similarity effectively. Inspired by temporal constraints on the original PPR (TPPR [12]), we discovered that such methods for node similarity representation can lead to more precise node embedding representations in temporal graphs. Based on the temporal attributes, this method represents the edges of the directed graph, which better represents the random walk sequence. Specifically, it constructs a temporal transition matrix by incorporating the connectivity status across different timestamps. TPPR addresses challenges such as handling dangling edges where the destination of a temporal edge is uncertain, which are not effectively managed by traditional PPR methods. The algorithm begins by identifying interacting edges at each timestamp within the temporal graph. Subsequently, we compute TPPR values for individual nodes across the entire temporal span. Utilizing dynamic programming algorithms, we propagate transition probabilities throughout the graph. At each timestamp, we calculate the probabilities of nodes reaching their connected nodes based on previously identified interaction edges, adjusted by the current timestamp’s transition probabilities. Finally, we derive the TPPR vector for each node and aggregate these vectors to construct the comprehensive TPPR matrix.

By directly performing Single Value Decomposition (SVD) and Nonnegative Matrix Factorization (NMF) matrix decompositions on the TPPR matrix, we obtained graph embeddings and corresponding embedding vectors. Subsequently, we conducted downstream tasks such as link prediction, node classification, and node clustering [13,14,15,16,17] on multiple dynamic datasets. We evaluated the task results using specific metrics, reflecting the effectiveness of TPPR-based temporal matrix decomposition. During experiments, we compared our algorithm with existing temporal graph representation learning algorithms, traditional temporal graph learning algorithms, and graph neural-network-based algorithms across various temporal datasets. Through comparison with metrics from experiments, our algorithm demonstrates superior overall performance on dynamic datasets compared to baseline methods.

## 2. Related Work

### 2.1. Static Graph Representation Learning

Static graph representation learning involves learning the structure of nodes, edges, and the entire graph from static graph data and effectively representing them using low-dimensional vectors. This technique, referenced in studies [18,19,20], finds widespread applications in fields such as social network analysis, bioinformatics, recommendation systems, and knowledge graphs. Graph embedding algorithms have evolved towards more scalable methods. Matrix factorization techniques approximate the decomposition of the adjacency matrix to obtain embeddings. Meanwhile, random-walk-based methods use Skip Gram to generate embeddings from sequences of random walks, capitalizing on the graph’s sparsity to reduce time complexity.

There are many existing static graph representation learning methods. Random-walk-based methods are relatively classic, such as DeepWalk [21], node2vec [22], LINE [23], and struc2vec [24]. However, most of these are algorithms that modify and improve random walk strategies based on DeepWalk; Static graph embedding models based on matrix factorization decompose the feature matrices of node associations and attribute information matrices (e.g., SVD), then fuse the decomposed attribute embedding and structural embedding to generate low-dimensional embedding of the nodes, such as HOPE [10] and SPE [25]. Additionally, there are methods based on graph neural networks, such as Graph Convolutional Networks (GCNs) and GATs [26]. However, static graph representation learning methods still have limitation due to a lack of temporal awareness.

### 2.2. Temporal Graph Representation Learning

Temporal graph representation learning is a representation learning method for dynamic or temporal graphs, dealing with data of nodes and edges changing over time. Temporal graph representation learning [27,28,29,30,31,32,33,34,35,36,37,38] has wide applications in various fields such as traffic network analysis, financial transactions, social networks, and bioinformatics. It enhances graph representation learning from a temporal perspective to address the shortcomings of static graph representation learning. Similarly, the current direction of temporal graph representation learning draws on the experience of predecessors and makes improvements in random walks, matrix factorization, and graph neural networks regarding temporal attributes.

In random walk methods, Sajjad et al. [39] divided the process of graph embedding generation into two steps: first, updating random walk sequences on dynamic graphs. Compared with directly starting random walks from scratch on static snapshots, the updating algorithm maintains the statistical characteristics of random walks. Then, given the embedding representation of the previous time step and the updated random walk sequence, the Skip-Gram model is employed to update the embedding representation. CTDNE [40], on the other hand, addresses the temporal properties of dynamic networks, capturing temporal dependencies using the Hawkes process to learn the temporal evolution characteristics of the network. By learning low-dimensional representations of nodes over continuous time, CTDNE can address link prediction and node classification problems in dynamic networks. Similarly, there are still methods that study temporal graph representation learning using the Hawkes process and enhance node representations by incorporating attention mechanisms on this basis [41,42]. In matrix factorization methods, dynamic graph methods based on matrix factorization use eigenvalue decomposition to construct high-order similarity matrices of graphs and then use matrix perturbation theory to update the dynamic information of graphs, such as DANE [43] and DHPE [44]. In dynamic graph neural network learning, dynamic graph models based on GNNs usually introduce a cyclic mechanism to update network parameters on the basis of static graph models, achieving the modeling of dynamic processes, and also ensuring that the generated low-dimensional embedding effectively retain the dynamic evolution information of the graph. Examples include DySAT [45]. However, the current research still lacks methods to address the challenging issues mentioned in Section 1 regarding node similarity.

## 3. Preliminaries

Given a temporal graph G(V,E,T), where *V* represents the set of *n* vertices in graph *G*, *E* represents the set of *m* edges in graph *G*, and *T* represents the collection of timestamp sequences where interactions occur in the graph, in order to facilitate the description of temporal edges, each edge is represented as two ordered edges in opposite directions. Specifically, *<u,v,t>* is represented as *<u,v,t>* and *<v,u,t>*, where *u* and *v* are nodes, and *t* is the timestamp. Here, *head(*$\overset{⃑}{e}$*)* denotes the head node of the temporal edge, *tail(*$\overset{⃑}{e}$*)* denotes the tail node, and *time(*$\overset{⃑}{e}$*)* denotes the timestamp of the temporal edge. You can gain a more intuitive understanding from the following equation:

$$\overset{⃑}{e_{u}^{out}}=\overset{⃑}{e}|\mathrm{head}(\overset{⃑}{e})=u\;\;\;\overset{⃑}{e_{u}^{in}}=\overset{⃑}{e}|(\mathrm{tail}\overset{⃑}{e})\;=\;u$$

The temporal graph maps nodes to a vector of dimension d through a learned function, under the condition that the sampled nodes satisfy the temporal reachability criterion, and where *d* ≪ |*V*|. $f:V\to Z,Z=\{z_{1},\ldots ,z_{n}\},z_{i}\in R^{d}$, where V represents the node matrix, Z represents the embedding matrix, n is the number of nodes, and the vector $z_{i}$ corresponds to the vector representation of node $v_{i}$.

## 4. Methodology

The graph representation learning algorithms based on TPPR proposed in this paper adds a time constraint compared to the PPR algorithm. It computes the time-constrained node similarity of this node, named as TPPR value, for each node, and then integrates these TPPR values into a matrix. This matrix undergoes SVD or NMF matrix decomposition to obtain graph embedding vectors. This section specifically analyzed four perspectives. Section 4.1 defines temporal random walks and temporal transition matrices, highlighting the shortcomings of existing static PPR as mentioned earlier. Section 4.2 and Section 4.3 analyze how we solve the issue of dangling edges to implement TPPR, explaining the combination of PPR with time to derive TPPR. Section 4.4 demonstrates how to obtain vectors through SVD and NMF matrix decomposition. Section 4.5 provides an analysis of the algorithm for TPPR matrix decomposition presented in this paper.

### 4.1. Temporal Random Walks and Temporal Transition Matrices

In a static graph *G (V,E)*, there are connections between different nodes. Calculating the similarity between nodes involves computing the personalized PageRank (PPR) [9] value for each node. The computation of PPR involves passing PPR to other connected users through random walks, starting from the initial PPR values. We can easily obtain the following equation:

(1) $$x=(1-\alpha )x_{0}+\alpha xM$$

(2) $$M_{ij}=\left\{\begin{array}{cc} \frac{1}{\left|out(i)\right|} & ,\;j\in out(i)\\ 0 & ,\;else \end{array}\right.$$

In the above formula, $x_{0}$ represents the initial distribution of PPR values, *x* represents the PPR distribution values, α represents the damping factor, M is the transition probability matrix, $M_{ij}$ represents the transition probability from node *i* to node *j*, and out(i) indicates the out-degree of node *i*, where *i* is accessible to *j*. Given the initial PPR values for each user, random walks are conducted with a probability of (1 − α) based on the existing user connections and damping factor.

In addressing the issue of temporal graphs, each node is regarded as a moving entity and moves within the graph through random walks. Unlike traditional static graphs, temporal graphs contain temporal information, so the connectivity and attributes of nodes in the graph may change over time. However, existing PPR random walks lack temporal coherence, and in a temporal graph, there may be multiple interactions between two points at different time points, which cannot be interpreted by the transition matrix M of PPR. Therefore, to more accurately characterize the similarity between nodes in temporal graphs, we introduced the concepts of temporal random walks and temporal transition matrices.

**Definition** **1** (Temporal Random Walks [12])**.** *Given that l-hop temporal random walk from node i to node j with an ordered list of edges, {$\overset{⃑}{e_{1}}$, $\overset{⃑}{e_{2}},\ldots ,\overset{⃑}{e_{l}}$}. This temporal sequence satisfies the conditions, head($\overset{⃑}{e_{1}})=i$, tail($\overset{⃑}{e_{l}}$) = j, and when 1 ≤ x ≤ l − 1, tail($\overset{⃑}{e_{x}}$) = head($\overset{⃑}{e_{x+1}}$) and time($\overset{⃑}{e_{x}}$) ≤ time($\overset{⃑}{e_{x+1}}$). Here, we use ${tw}_{l}$ and ${TW}_{l}^{u\to v}$ as sets of l-hop temporal walks and temporal walk edges from u to v, respectively.*

**Definition** **2** (Temporal transition matrix [12])**.** *Temporal transition matrix between two temporal edges **P**∈$R^{m\times m}$ can be computed as follows:*

(3) $$P(\overset{⃑}{e_{i}}\;\to \overset{⃑}{e_{j}})=\left\{\begin{array}{ll} \frac{g(time(\overset{⃑}{e_{j}})-time(\overset{⃑}{e_{i}}))}{\sum_{\vec{e_{k}}\in N>(\vec{e_{i}})}g(time(\vec{e_{k}})-time(\vec{e_{i}}))} & ,\vec{e_{j}}\in N>(\vec{e_{i}})\\ 0\; & ,\vec{e_{j}}\notin N>(\vec{e_{i}}) \end{array}\right.$$

In this formula, $\left(\overset{⃑}{e_{i}}\;\to \overset{⃑}{e_{j}}\right)$ denotes the transition probability from $\overset{⃑}{e_{i}}$ to $\overset{⃑}{e_{j}}$, while $N>(\vec{e})=\{<u,v,t>|u=tail(\vec{e}),t>time(\vec{e})\}$. Here, *g(a,b)* is a decay function used to capture the dependency between connections, and $\vec{e_{j}}$ should satisfy the set of $N>(\vec{e_{i}})$. In this case, we use a linear decay function (other functions such as exponential or logarithmic functions can also be used). It is noteworthy that P matrix can only be constructed once for each dataset to perform TPPR calculation. For the l-hop temporal walk ${tw}_{l}$ = {$\overset{⃑}{e_{1}}$, $\overset{⃑}{e_{2}},\ldots ,\overset{⃑}{e_{l}}$}, the transition probability of the l-hop temporal walk ${tw}_{l}$ is indicated by *P(*${tw}_{l}$). Specifically, *P(*${tw}_{0}$*) =* 0, and when ${tw}_{l}\;$*= <u,v,t>*, *P(*${tw}_{l}$*) =* 1*/|*$\overset{⃑}{e_{u}^{out}}$*|*.

### 4.2. Handling of Danging Edges

Unlike static graphs, edges in temporal graphs may change over time, leading to uncertain reachability, which means edges in this state cannot reach any connected edges at the current time. We call this state as “dangling state”, and edges in this state are referred to as “dangling edges”. This condition can be expressed using transition probabilities: ${\sum_{l}^{out({\vec{e}}_{i})}P}_{{\vec{e}}_{i}}({\vec{e}}_{i}\to {\vec{e}}_{j})=0$. To simplify this formula, we have $P_{{\vec{e}}_{i}}({\vec{e}}_{i}\to {\vec{e}}_{i})=1$, indicating the dangling state. This ensures that the temporal transition matrix P is a stochastic matrix.

Dealing with dangling edges is an inevitable challenge in the context of temporal graphs. Therefore, when conducting random walks, we take into account the influence of dangling edges. Combined with Definitions 1 and 2, we provide the following definition:

**Definition** **3** (l-hop Temporal transition matrix)**.** *For l, if there exists an integer l satisfying (i) when 1 ≤ i ≤ l − 1, time($\vec{e_{l}})<$ time($\vec{e_{l+1}})$ and $\vec{e_{l}}$ not a dangling state, (ii) $\vec{e_{l}}$ is a dangling state and for all integers k, $\vec{e_{l}}=\vec{e_{l+k}}$, then, we consider $P_{tw_{\infty }}\neq 0$.*

### 4.3. Time-Constrained Personalized PageRank

**Figure 2:**
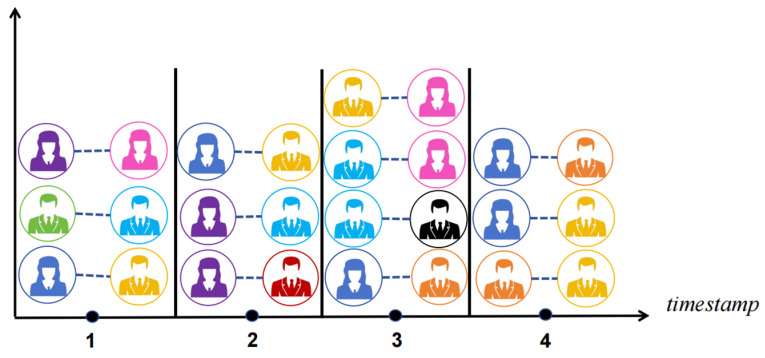
Edge stream based on dynamic social Figure 1.

In a temporal graph, the connections between nodes evolve over time. Therefore, to characterize the similarity between nodes within a temporal graph, we calculate Time-constrained Personalized PageRank (TPPR) by adding a time constraint to obtain a time-constrained transition matrix compared with the PPR algorithm. This step is a key point of the algorithm proposed in this paper. Here, we introduce edge streams to represent the graph, describing the interactions occurring within each timestamp. This assists us in extracting information from the temporal graph, as shown in Figure 2.

**Definition** **4** (TPPR [12])**.** *Given a dynamic graph G(V,E), node q, and damping factor α, the computation of a node’s TPPR relative to node q under time constraints can be represented as follows:*

(4) $$tppr_{q}(u)=\sum_{\vec{e}\in {\vec{e}}_{u}^{in}}\tilde{ppr}(\alpha ,{\tilde{X}}_{q})(\tilde{e})$$

According to Equation (1), we can equivalently obtain:

(5) $$\tilde{ppr}(\alpha ,\tilde{X_{q}}=\alpha \tilde{X_{q}}+(1-\alpha )\tilde{ppr}(\alpha ,\tilde{X_{q}})P$$

If $\tilde{X_{q}}\in R^{1\times m}$ and $\vec{e}\in \vec{e_{q}^{out}},then\tilde{X_{q}}(\vec{e})=1/|\vec{e_{q}^{out}}|$.

Unlike the random walk process in traditional static PPR, computing temporal TPPR involves traversing ordered temporal edges, taking into account not only the connectivity of nodes but also the times at which these connections occur. (i) $\tilde{ppr}\;(\alpha ,\tilde{X_{q}})(\vec{e})$ reflects the temporal similarity of $\vec{e}$ to point *q*. (ii) Since P is a stochastic matrix and $\tilde{ppr}(\alpha ,\tilde{X_{q}})$ is probability distribution, we can thus easily obtain the equation to calculate the TPPR value related to node *q* as $\sum_{u}{tppr}_{q}(u)=\sum_{u}\sum_{\vec{e}\;\in \vec{e_{u}^{in}}}\tilde{ppr}(\alpha ,\tilde{X_{q}})(\vec{e})=1$. The TPPR value is also a probability distribution, so it is reasonable to interpret the temporal correlation between point *u* and point *q* with ${tppr}_{q}$. After simplification, we use tppr(u) to represent ${tppr}_{q}(u)$ in the subsequent parts.

**Lemma** **1** ([12])**.** *Given a temporal graph G(V,E,T), a node q, and a transition probability α, we have [Proof see [12]]*

(6) $$tppr(u)=\sum_{i=0}^{\infty }{\alpha (1-\alpha )}^{i}\sum_{{tw}_{i+1}\in {TW}_{i+1}^{q\to u}}P(tw_{i+1})$$

According to Lemma 1, a relatively simple approach is enumerating all timely sequential walks from node *q* to any node *u* at first, then calculating the transition probabilities from node *q* to node *u* by considering the previous time-sequential walks, and finally obtaining tppr(u) through Lemma 1. However, since the sum of the probabilities tends to infinity, it is impossible to accurately compute tppr(u). Hence, directly applying Lemma 1 to compute tppr(u) is challenging. 

When considering the dangling state and Definition 4, we assume that the time-sequential walks based on probability α satisfy (i) that they start from node q and (ii) the probability of stopping at the current state at each step being α, and continuing to walk with a probability of (1 − α) and Equation (4). Additionally, we use $u^{t}$ to denote any ordered time-sequential edge $\vec{e}$, where tail($\vec{e}$) = *u* and time($\vec{e}$) = *t*. Let D[u][t] represent the probability that the time-sequential walks stop at $u^{t}$ in a given time-sequential walk, with at most one dangling state (if any).

**Lemma** **2** ([12])**.** *Given a temporal graph G(V, T), a node q, and a transition probability α, we have: $tppr(u)=\sum_{t\in T_{1}}D[u][t]+\sum_{t\in T_{2}}D[u][t]/\alpha$, $T_{1}$ = {$t|u^{t}\;$ is not a dangling state}, $T_{2}$ = {$t|u^{t}\;$ is dangling state}. [Proof see [12]].*

Based on Lemma 2, we derived a method to compute the TPPR for each qualified node u, which can also be describe as *tppr(u)*. We define the algorithm’s input and output as the temporal graph and the transition probability α, respectively. Following that, we initialize the TPPR values for each node related to other nodes as 0, and intermediate variables, *D*[*u*], are also initialized to 0.

The first step involves generating an edge stream representation graph based on the temporal graph. Then comes steps which are similar to PPR. We propagated the transition probabilities to the entire graph. Following the dynamic programming paradigm and the previous generated edge stream representation graph, we calculated the probability of reaching node *u* at each time point. Then, we multiplied this probability by the probability of transitioning from the current time to node *v* found in the transition probability matrix. If the starting point of the currently calculated edge was the same as the node where the TPPR value was being calculated, then the probability of transitioning to node *v* was determined by adding the current transition probability to the transition probability divided by the out-degree of node *u*. Following that, we added up all the transition probabilities at each time to obtain the TPPR value for the current node. Above all, we computed the TPPR vector for each node and combined them all to form the complete TPPR matrix. This matrix can then be used for downstream tasks through subsequent matrix decomposition. We can see algorithm for TPPR more clearly from Algorithm 1:
**Algorithm 1** Compute *tppr*(*G,q,α*) [12]INPUT: temporal graph G, transition probility αOUTPUT: uϵV, compute u’s tppr value related to *q*
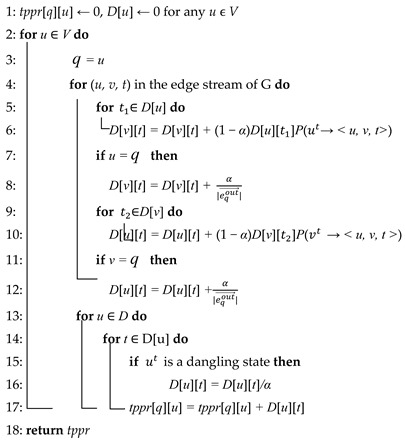


### 4.4. Matrix Factorization

After these steps, we obtained the TPPR matrix containing the transition probabilities of all nodes relative to each other, and decomposing the node similarity matrix is a classic method in graph representation learning. The next task is to conduct two classical matrix decomposition methods, Single Value Decomposition (SVD) and Nonnegative Matrix Factorization (NMF), to decompose the TPPR matrix and obtain embeddings. Additionally, we use both methods in our experiments and compare and discuss the different effects of the matrix decomposition.

#### 4.4.1. TPPR-SVD

SVD is a classical matrix decomposition operation that is widely used for embedding techniques to address high-dimensional datasets. It decomposes a larger matrix into three different matrices multiplied together: a unitary matrix, a diagonal matrix, and another unitary matrix. Each matrix contains distinct information and is extensively utilized in various scenarios such as image compression, image denoising, and pattern recognition. When SVD decomposition is applied to text information extraction, it decomposes a matrix describing the association between articles and words. The first resulting matrix represents the categorization of words with rows representing words and columns representing semantic classes, the second matrix depicts the association between word classes and article classes, and the last matrix signifies the categorization results of articles with each column representing an article and each row representing a topic. By analogizing text information extraction to graph information extraction, similar effects can also be achieved (Algorithm 2).

**Definition** **5** (SVD)**.** *Given a k-dimensional decomposition of matrix A, and the corresponding singular vectors, we can obtain the embedding, $\left[f(\sigma_{1})u_{1},\ldots ,f(\sigma_{k})u_{k}\right]$, where f(•) is the weighted function of singular values.*

(7) $$A\overset{SVD}{\Rightarrow }\sum_{\;i=1}^{N}\sigma_{i}u_{i}^{s}{v_{i}^{t}}^{T}$$

where a hyper-parameter is typically set to the value of $\frac{1}{2}$, commonly in graph representation learning. This yields the following embedding, $\left[\sigma_{1},\ldots ,\sigma_{k}\right]$, which is the singular values sorted in descending order; $\left[\sigma_{1}u_{1},\ldots ,\sigma_{k}u_{k}\right]$ represents the coordinates of the data subspace, and $\left[v_{1},\ldots ,v_{k}\right]$ represents the projection of the data onto the signal subspace.

(8) $$U^{s}=[\sqrt{\sigma_{1}}\cdot u_{1}^{s},...,\sqrt{\sigma_{k}}\cdot u_{k}^{s}]$$

(9) $$U^{t}=[\sqrt{\sigma_{1}}\cdot v_{1}^{t},...,\sqrt{\sigma_{k}}\cdot v_{k}^{t}]$$

**Algorithm 2** TPPR-SVD INPUT: Matrix TPPR, embedding dimension *k* OUTPUT: embedding vector $U^{s}$, targeting vector $U^{t}$1: Compute $M_{g},M_{l}$, $M_{g\;}=I-\alpha P$, $M_{l}=(l-\alpha )\cdot I$2:Perform np.linalg.svd, a SVD method provided by numpy, on $M_{g},M_{l}$ to obtain the generalized singular vectors {$\sigma_{1}^{l}$, …, $\sigma_{k}^{l}$} and {$\sigma_{1}^{g}$, …, $\sigma_{k}^{g}$}, long with the corresponding singular vectors {$v_{1}^{s}$, …, $v_{k}^{s}$} and {$v_{1}^{t}$, …, $v_{k}^{t}$}3:Calculate the singular values {$\sigma_{1}$,...,$\sigma_{k}$} according to Equation (7).4:Calculate the embedding matrix $U^{s}$ and $U^{t}$ according to Equations (8) and (9).

#### 4.4.2. TPPR-NMF

NMF is similar to SVD, but it incorporates a non-negativity constraint, ensuring that all elements of the decomposed matrices are non-negative. Non-negativity enhances the relevance of data, as negative values are irrelevant to the dataset. This characteristic makes NMF particularly suitable for datasets with only non-negative signals, such as pixel values in images, word frequencies in text, and so on. In recommendation systems, NMF provides a more natural interpretation of the decomposed factors, such as users’ latent interests or products’ latent attributes. NMF decomposes a matrix into the product of two non-negative matrices, which enables the discovery of linear representations of non-negative components. In graph representation learning, NMF can be employed to decompose adjacency matrices or other network feature matrices, thus obtaining low-dimensional representations of nodes (Algorithm 3).

**Definition** **6** (NMF)**.** *For a matrix $A_{m*n}$, using NMF for matrix factorization decomposes A into the product of two matrices, $W_{m*k}$ and $H_{k*n}$, both non-negative. Specifically, k<min(m,n) and it takes the form,A≈W∗H.*

To find suitable matrices, W and H, we need to optimize a loss function, such as the square error function (Frobenius norm) or KL divergence. The square error loss function is represented as $L={\left\|A-W*H\right\|}_{F}^{2}$, where ${\left\|.\right\|}_{F}$ denotes the Frobenius norm. By iteratively applying optimization algorithms like gradient descent or alternating least squares to minimize the loss function L, we can find the approximate non-negative matrices *W* and *H* that represent *A*.

In this work, the embedding vectors obtained from the NMF decomposition of the TPPR matrix are the row vectors in matrix *W.* As defined in Definition 6, k<min(m,n), this decomposition achieves dimensionality reduction of the matrix. In the context of NMF, embedding vectors represent the original data in a lower-dimensional space. Each row in matrix *W* corresponds to a data point (e.g., a node in a graph) in the original data matrix *A*. These row vectors (i.e., embedding vectors) adeptly capture the local structure and patterns of the original data, mapping them to a lower-dimensional space. This low-dimensional representation facilitates the extraction of useful features for subsequent machine learning tasks such as classification, clustering, and prediction.
**Algorithm 3** TPPR-NMFINPUT: Matrix TPPR, embedding dimension *k* OUTPUT: Matrix *W*1: Initialize parameters, select the dimensionality *k* of the low-dimensional space, and initialize matrices *W* and *H*.2: Convergence check: Examine whether the objective function has converged or reached the maximum number of iterations; if so, stop the iterations3: Extract embedding vectors: Utilize the row vectors of matrix *W* as low-dimensional embedding vectors for subsequent machine learning tasks and data analysis.

### 4.5. Complexity Analysis

In this section, we analyze the time and space complexity of the Algorithms 1, 2 and 3.

- TPPR: The algorithm’s time complexity primarily arises from the computation of the TPPR matrix, which involves three nested for loops. The first for loop iterates over all nodes in the graph, with time-complexity of O(V). The second for loop iterates over the edge flow representation graph, with a time-complexity of O(E). The third for loop iterates over the timestamps in the *D[u]*, with a time- complexity of O(V∗E∗T). Therefore, the overall time complexity of the algorithm is O(V∗E∗T). Additionally, the algorithm requires space to store the TPPR values of each node, resulting in a space complexity of $O(V^{2})$.
- SVD: The primary time expense in performing SVD matrix decomposition lies in decomposing TPPR. This involves calling the np.linalg.svd (tppr) function from the numpy library, with a time complexity of $O(\min(m^{2}*n,m*n^{2}))$, where m and n represent the number of rows and columns of the TPPR matrix, respectively. Since TPPR is a matrix (with columns and rows both of size V), the time complexity of SVD is $O(V^{3})$. In terms of space complexity, the main overhead occurs in storing the left k singular vectors, U_k=U[:,:k], and the first k rows and columns of the singular value matrix Σ, as well as the first k non-zero singular values and the elements on the diagonal, sqrt_sigma_k=sqrt_sigma[:k,:k]. The space complexity is O(V∗k).
- NMF: In the Scikit-learn library, NMF uses the coordinate descent optimization algorithm, with a time complexity of approximately O(t∗n_components∗(m+n)), where t is the number of iterations; in this case, n_components equals to k. Therefore, the time complexity is O(t∗k∗(m+n)). The space complexity mainly depends on the matrices W and H. The size of matrix W is m∗n_components, and the size of matrix H is n∗n_components, resulting in a space complexity of O(k∗(m+n)).

## 5. Experimental Setup

To further evaluate the performance of our algorithm, we conducted experiments on seven real-world available dynamic graph datasets and compared our algorithms with other six baseline methods in three directions: node classification, link prediction, and node clustering.

### 5.1. Dataset

The *soc-sign-bitcoinotc* dataset contains anonymous Bitcoin transaction information from the Alpha Bitcoin website; ***Soc-sign-bitcoinotc*** holds anonymous information exchange data from the OTC Bitcoin table trading website; ***Soc-wiki-elec*** collects election data for ***Wikipedia*** administrators, including historical voting data for administrators after the last ***Wikipedia*** page edit (since 3 January 2008); the ***Escorts*** dataset gathers data on transactions for a certain commodity, where “1” represents positive feedback, “0” represents neutral feedback, and “−1” represents negative feedback; ***Email-dnc*** contains email information from the time of the 2016 United States Democratic National Committee email leak, recording email transmission information among individuals; ***Fb-forum*** records activity information of individuals in social media forums similar to Facebook; and ***IA-contact*** is a database simulating human social behavior [46]. All of these databases show strong interactions over time, making them typical temporal graphs, which is suitable for our task. By applying our algorithm to such datasets for experiments, we can obtain more accurate results seen in Table 1.

### 5.2. Baseline Method

We compare our algorithms TPPR-S and TPPR-N, in reference to the SVD method and the NMF method applied to the TPPR matrix respectively, with baseline methods, Spe [25], CTDNE [40], Dynamicnode2vec [47], PMLP [48], ARMA [49], and FAC [50]. Among them, PMLP, ARMA, and FAC are the methods within the framework of deep learning, while Spe, CTDNE, and Dynamicnode2vec are traditional graph representation methods. Our task is to compare our algorithm with these classical dynamic graph algorithms and latest algorithms.

- SPE [25]: This algorithm generates node embedding vectors by perturbing the graph structure, introducing perturbations for each node to alter edge relationships. Node embedding is computed based on the perturbed structure, capturing node neighborhoods and positions. For temporal graphs, feature decomposition based on time intervals and sub-graph yields embedding. SPE aims to evaluate whether matrix factorization outperforms traditional methods on temporal graphs.
- CTDNE [40]: CTDNE learns embedding representations with time information from continuous dynamic graphs using temporal random walks. It follows static graph methods but orders random walks by edge occurrences’ timestamps. Time information reduces embedding uncertainty, thereby enhancing CTDNE’s performance over Deep-walk [21] and node2vec [22] on diverse tasks.
- Dynnode2vec [47]: It utilizes node2vec [22] for embedding and Skip-Gram training on snapshot G1. For subsequent snapshots, it uses dynamic Skip-Gram and generates random walk sequences for evolving nodes. Embedding from the previous moment serves as initial weights, updated with random walks of evolving nodes for the current moment. By focusing on evolving nodes, dynnode2vec enhances model efficiency as most node neighborhoods remain unchanged in gradual graph evolution.
- PMLP [48] is an intermediate model that builds upon traditional Graph Neural Networks (GNNs) by incorporating Propagation MLP (PMLP), achieving higher training efficiency while retaining the ability of traditional GNNs to effectively capture complex graph structure features in node prediction tasks.
- ARMA [49] introduces a novel graph convolutional layer that is inspired by the auto-regressive moving average (ARMA) filter, offering a more flexible frequency response compared to polynomial filters. This algorithm incorporates ARMA filters in a recursive and distributed form to obtain an effective training convolutional layer, resulting in significant improvements in training outcomes.
- FAC [50] explores the learning effects of low-frequency information to investigate whether GNNs can adaptively learn more information. Based on this, we propose a novel Frequency Adaptation Graph Convolutional Network (FAGCN), which can adaptively integrate different signals and conclude that the distinction between high and low frequencies has a significant effect on graph representation learning.

### 5.3. Node Classification

**Figure 3:**
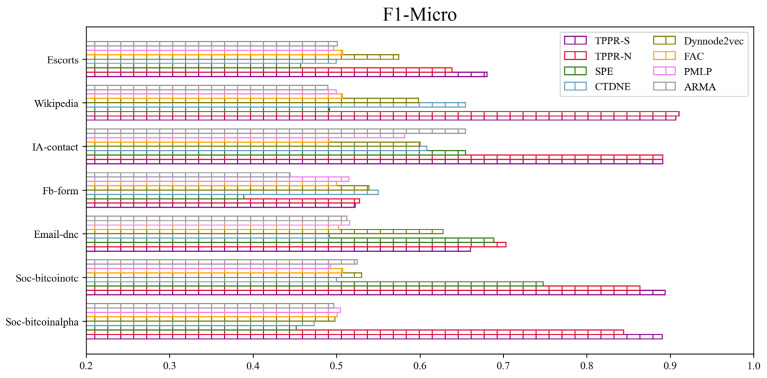

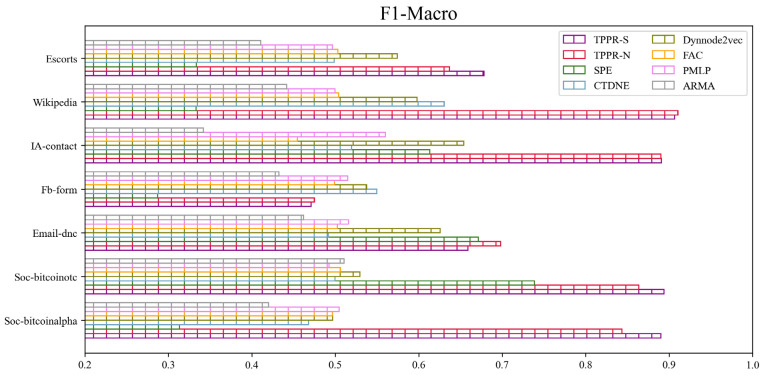
Node classification performance of different baseline methods in different datasets.

**Figure 4:**
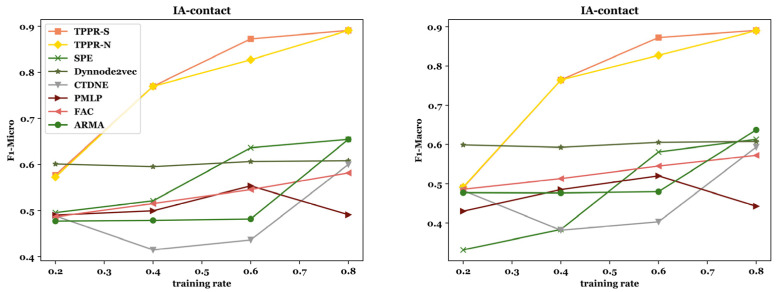

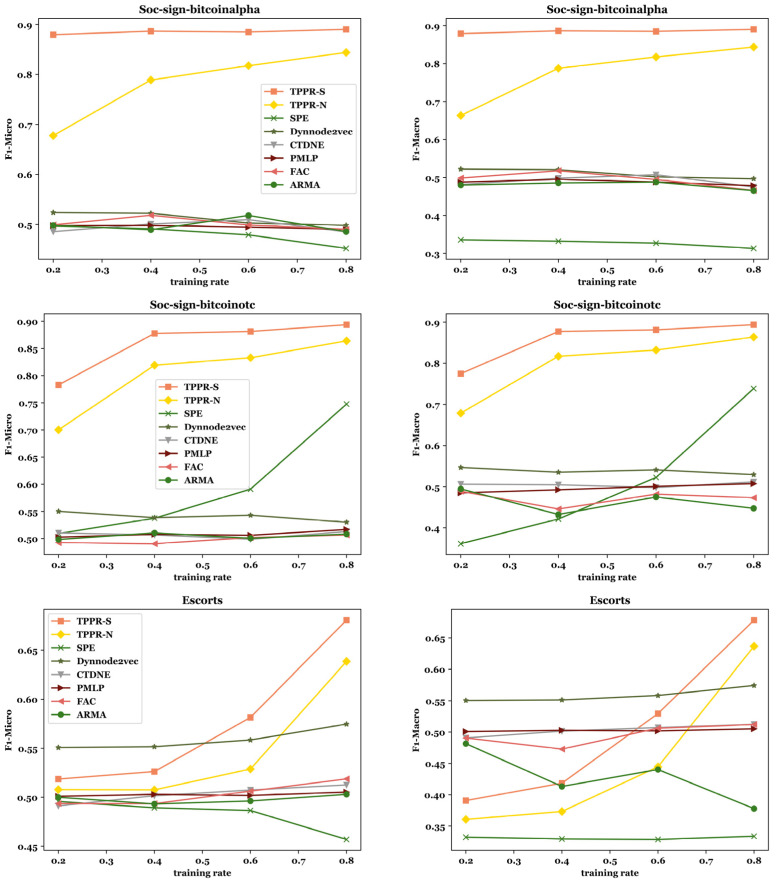
F1-micro and F1-macro by changing training rate in *IA-contact, Soc-sign-bitcoin-alpha, Soc-sign-bitcoinotc,* and *Escorts*.

Node classification aims to categorize nodes into different labels and classes based on node attributes and connectivity relationships. In this task, the objective is to predict the category to which nodes belong using the network topology and node attribute information. Through selectingnodes for training witha logistic regression classifier, we evaluate the remaining nodes to obtain Micro-F1 and Macro-F1 scores. After training the baseline methods and our algorithms TPPR-S and TPPR-N on seven different datasets and evaluating Macro-F1 and Micro-F1 metrics, the scores of TPPR-S and TPPR-N get superior overall performance in Figure 3. To achieve more accurate and unbiased results in the node classification task, we investigat the training rate in the node classification task. By varying the training rate α from 20% to 80% in the node classification task, we observe that higher training proportions leads to better performance. To mitigate errors caused by dataset partitioning, we repeat the calculations 20 times in each experiment, averaging the results after removing outliers. The corresponding data are presented in Figure 3 and Figure 4.

From Figure 3, among the experiments conducted on seven datasets, our TPPR-S and TPPR-N algorithms exihibit superior performance, achieving the best results in six of them and both of them achieving the best results in five of them based on F1-Micro and F1-Macro metrics. In particular, in financial datasets like *Soc-sign-bitcoinalpha* and *Soc-sign-bitcoinotc*, our metrics outperform those of the other five baseline algorithms by nearly 80%. In the experiments through varying the training set ratio parameter, the result shown in Figure 4 indicates that a higher training set ratio leads to superior evaluation metrics. Therefore, for a more concise analysis, we choose 80% as the training ratio with the best metrics for node classification experiments. Overall, we can conclude that our algorithm continued to outperform the other six baseline in terms of overall performance when selecting the optimal training rate.

### 5.4. Link Prediction

Link prediction means finding connections that may exist but have not yet been observed with the existing network topology. In this experiment, we combine node embedding representations into edge embedding representations using an operate function. Then, we transform node embedding representations and edge information into a dataset for the link prediction task, where positive edge labels are assigned 1 and negative edge labels are assigned 0. After training through a logistic regression model on the preprocessed training dataset, we can evaluate the trained model on the test set to obtain precision metrics. To ensure accuracy in the experimental data, each precision metric is trained 20 times and then the averaged-number served as experimental data (Table 2).

Compared the baseline methods with TPPR-S and TPPR-N, and evaluating using precision scores, we find from Table 3 that our algorithms exhibit better performance on the aforementioned datasets. Although Dynnode2vec performs better than our algorithms in link prediction results for datasets *Fb-forum*, *IA-contact,* and *Wikipedia*, across these seven datasets, our scores in the link prediction task are higher than Dynnode2vec’s. Moreover, through comparison, it is found that the TPPR-S algorithm outperforms TPPR-N in link prediction.

### 5.5. Node Clustering

We employ the K-means [51] algorithm for node clustering experiments. K-means clustering is a widely used method that partitions data points into K clusters, with each cluster’s centroid being the mean of all its members. The algorithm iteratively seeks the optimal cluster centers until convergence is achieved. In our experiment, we use K-means to generate 10 clusters from node vectors and obtained corresponding cluster labels. We evaluate the quality of node clustering using two metrics: Silhouette score [52] and Davies–Bouldin index (DB) [53]. These metrics respectively measure the ratio of the distance of each sample point to its intra-cluster samples versus the inter-cluster distance and the ratio of within-cluster distance to between-cluster distance. The results for node clustering are presented in Table 3.

From the data, we conclude that our method comprehensively outperformed Dynamicnode2vec and FAC algorithms. However, it is also evident that the Silhouette scores of CTDNE and PMLP methods are superior to TPPR-S and TPPR-N algorithms across all datasets, except the escorts dataset. Notably, TPPR-S achievesthe highest Silhouette value among all algorithms, specifically in the escorts dataset, indicating significant potential for TPPR-S in certain specific datasets. In terms of Davies–Bouldin (DB) index values, our methods perform better than CTDNE and PMLP in three out of seven datasets, comparable to these algorithms in others. Considering the comprehensive analysis across node clustering experiments, our algorithm demonstrate some limitations in this aspect. However, given our investigation across three distinct experiment types—link prediction, node clustering, and node classification—it is essential to assess the overall performance of algorithms. Despite not excelling in node clustering experiments, our methods consistently outperform others in node classification and link prediction experiments across more than 80% of datasets on average. Therefore, when considering all aspects together, our TPPR-S and TPPR-N algorithms produce superior embeddings, establishing them as advantageous approaches.

## 6. Conclusions

In this work, we propose a temporal graph representation learning framework based on TPPR matrix factorization. By using TPPR as a time-constrained method that can calculate the node’s proximity in temporal graphs, we are able to capture the structural characteristics of temporal graphs better. Additionally, to improve the calculation of node similarities in temporal graphs, we introduce the concept of temporal transition matrix, which enables a more effective capture of temporal information. The experimental results demonstrate the effectiveness of our proposed method in tasks such as node classification, link prediction, and node clustering. This is sufficient evidence to demonstrate that our proposed algorithmic framework is more effective and accurate in addressing the problem of dynamic graph representation learning compared to other methods.

## Figures and Tables

**Table 1:** Statistics of datasets.

Dataset	$\left|V\right|$	$\left|E\right|$		Timestamps	Self- Tmax
		Dynamic Edge	Static Edge		
***Soc-sign-bitcoinotc***	5881	35,579	21,492	35,445	1298
***Soc-sign-bitcoinalpha***	3783	16,863	14,124	1647	494
***Soc-wiki-elec***	7115	106,985	100,753	101,012	1341
***Escorts***	10,106	50,412	39,016	1913	448
***Fb-forum***	899	33,682	7036	33,483	1840
***IA-contact***	274	28,226	2124	15,662	1529
***Email-dnc***	1866	31,725	4384	18,682	4122

**Table 2:** Link prediction results on precision scores. (The bolded numbers are the ones with the highest scores in the same dataset).

Algorithm	*Soc-Bitcoinalpha*	*Soc-Bitcoinotc*	*Email-dnc*	*Fb-Forum*	*IA-Contact*	*Wikipedia*	*Escorts*
TPPR-S	**0.67521**	**0.58316**	**0.67897**	0.56097	0.82481	0.52980	**0.56328**
TPPR-N	0.64506	0.58196	0.66512	0.55719	0.83285	0.51581	0.54841
SPE	0.50674	0.52168	0.57971	0.51578	0.67589	0.52887	0.49567
CTDNE	0.50532	0.50415	0.49069	0.50000	0.49763	0.49467	0.49759
Dynnode2vec	0.60909	0.58120	0.66312	**0.61523**	**0.86627**	**0.56693**	0.54000
FAC	0.4943	0.5031	0.5123	0.5514	0.5181	0.5018	0.4928
PMLP	0.6225	0.5382	0.6637	0.6008	0.6623	0.5899	0.5363
ARMA	0.5001	0.5	0.5	0.5	0.5	0.5	0.5

**Table 3:** Node clustering results on precision scores.

Silhouette/DB	*Soc-Bitcoinalpha*	*Soc-Bitcoinotc*	*Email-dnc*	*Fb-Forum*	*IA-Contact*	*Wikipedia*	*Escorts*
TPPR-S	0.2914/ **0.5321**	0.1156/ 1.9308	0.2466/ 1.6749	0.1058/ 1.0776	0.1720/ 1.3512	0.2236/ 0.6366	**0.7157/** **0.4076**
TPPR-N	0.1270/ 0.7531	0.1729/ 1.0399	0.2514/ 1.1206	0.0503/ 1.0990	0.2237/ 1.0853	0.3085/ **0.5085**	0.1560/ 0.7870
CTDNE	0.3122/ 1.1524	0.2952/ 1.2414	0.3736/ 0.9168	**0.3569/** 0.9248	0.4265/ **0.6743**	**0.4229/** 1.3990	0.3044/ 1.3172
Dynnode2vec	0.0695/ 3.1459	0.0796/ 3.0318	0.2409/ 1.8619	0.0295/ 4.1131	0.0856/ 2.5546	0.0846/ 2.5961	0.0470/ 3.6478
FAC	0.0896/ 2.1195	0.0830/ 2.2144	0.0927/ 2.0176	0.0692/ 2.1991	0.0857/ 1.9859	0.0825/ 2.1231	0.0845/ 2.1877
PMLP	**0.3469/** 0.8371	**0.3060/** **0.8715**	**0.4428/** **0.8579**	0.3237/ **0.9091**	**0.4661/** 0.7063	0.3808/ 0.8573	0.3038/ 0.8482

## Data Availability

The raw data supporting the conclusions of this article will be made available by the authors on request.

## Abbreviations

**Notation**	**Description**
G(V,E)	*V* denotes the set of *n* vertices within graph *G* and *E* denotes the set of *m* edges in graph *G*
α	Damping factor, the probability that a node remains stationary during the random walk process
$M,M_{ij}$	Transition probability matrix, the probability of transitioning from node i to node j
$\overset{⃑}{e}$	Temporal edge
P	Temporal probability transition matrix
$P\left(\overset{⃑}{e_{i}}\to \overset{⃑}{e_{j}}\right)$	Transition probability from $\overset{⃑}{e_{i}}$ to $\overset{⃑}{e_{j}}$
q,u	Related node
$\overset{⃑}{e_{u}^{out}},\overset{⃑}{e_{u}^{in}}$	Head node and tail node of a temporal edge
${tw}_{l}$	The *l*-hop temporal random walk sequence
$X_{0}$	Initial distribution of static PPR
$X_{0}$	Initial distribution of temporal PPR
$u^{t}$	Node *u* at time *t*
